# Reconsidering patient‐centred care: Authority, expertise and abandonment

**DOI:** 10.1111/hex.13815

**Published:** 2023-07-20

**Authors:** Alison Pilnick

**Affiliations:** ^1^ School of Sociology and Social Policy University of Nottingham Nottingham UK

**Keywords:** abandonment, authority, choice, control, expertise, patient‐centred care

## Abstract

Patient‐centred care is commonly framed as a means to guard against the problem of medical paternalism, exemplified in historical attitudes of ‘doctor knows best’. In this sense, patient‐centred care (PCC) is often regarded as a moral imperative. Reviews of its adoption in healthcare settings do not find any consistent improvement in health outcomes; however, these results are generally interpreted as pointing to the need for more or ‘better’ training for staff, rather than raising more fundamental questions. Patient autonomy is generally foregrounded in conceptualizations of PCC, to be actualized through the exercising of choice and control. But examining healthcare interaction in practice shows that when professionals attempt to enact these underpinnings, it often results in the sidelining of medical expertise that patients want or need. The outcome is that patients can feel abandoned to make decisions they feel unqualified to make, or even that care standards may not be met. This helps to explain why PCC has not produced the hoped‐for improvement in health outcomes. It also suggests that, rather than focusing on scoring individual consultations, we need to consider how medical expertise can be rehabilitated for a 21st century public, and how patient expertise can be better incorporated into co‐design and co‐production of services and resources rather than being seen as something to be expressed through a binary notion of control.

**Patient and Public Contribution:**

This viewpoint draws on research conducted by the author across a range of settings in health and social care, all of which incorporated patient and public involvement when it was conducted.

## 1 BACKGROUND

‘Patient‐centred care’ (PCC) is a term first adopted by the psychoanalyst Michael Balint in the 1950s, in his work with English General Practitioners.^1^ However, Balint sometimes used other terms, such as ‘patient‐oriented’, and even at the outset, definitions were tricky; he defined it largely in terms of what it was not‐ the strictly biological, reductionist approach of illness‐centred medicine—rather than what it was. Balint was an advocate for holism, invoking the ‘pathology of the whole person’; in accordance with his psychoanalytic background, his emphasis was very much on relational matters. However, he also stressed to the practitioners that he worked with that they were participants in a ‘lop‐sided’ relationship, because of the asymmetrical distribution of medical knowledge between patient and doctor, and the fact that the patient sought consultations because they were unable to understand or resolve medical problems independently. The first empirical application of the concept of PCC was in the 1970s, in Patrick Byrne and Barrie Long's work; they audio recorded the consultations of 60 UK GPs.^2^ Analytically, they drew distinctions between doctor or patient‐centred behaviours, with the implication that patient‐centred behaviours were to be aspired to and doctor‐centred ones avoided. An example of this analysis in practice is that asking broad questions was seen as patient‐centred, whereas closed ones were seen as doctor‐centred.

Beginning contemporaneously with Byrne and Long's work, the 1970s and 1980s also saw the development of highly influential sociological work focused on the doctor/patient relationship, with sociologists such as Elliot Mishler and Howard Waitzkin conceptualizing the practice of medicine as a conflict or a struggle, through which patients were suppressed. This work brought ideas about medical paternalism to a wider audience, highlighting it as a problem that needed to be solved. And from the 1980s onwards, patient‐centred medicine began to be promoted both as an approach in its own right, rather than as a feature of other approaches, and as the way to address this problem of conflicting agendas between doctor and patient. The specific approach was developed by Joseph Levenstein and colleagues working in the Family Medicine Department at the University of Western Ontario in Canada^3^; for Levenstein and colleagues, patient‐centredness is a clinical method to address conflict.

## 2 HOW IS PATIENT‐CENTRED CARE DEFINED NOW?

Since the 1980s, there have been a range of attempts to further specify, define and measure PCC, but without any clear consensus. While there are now a variety of definitions and measuring tools (the UK charity The Health Foundation says there are more than 160), what seems to unite them is an emphasis on the importance of a transfer of control from doctor to patient. This is seen as a necessary counter to the problem of medical paternalism, as exemplified by the historical attitude that ‘doctor knows best’. However, as researchers have shown, commonly used measurement tools can produce quite different results as to whether the same healthcare consultation can be judged patient‐centred or not,^4^ which casts some doubts on their utility.

## 3 PATIENT‐CENTRED VERSUS PERSON‐CENTRED?

It is worth noting here that the term patient‐centred is sometimes used interchangeably with the term ‘person‐centred’. For example, updates in UK health policy documents have sometimes replaced the former term with the latter, without any other changes. However, the terms have different roots: ‘person‐centred’ originates in the work of the psychologist Carl Rogers and describes a particular approach in psychotherapy. Person‐centred therapy gets its name from the fact that its focus is on the client's subjective view of the world. But as the sociologist Nikolas Rose has illustrated so well, vocabularies taken from therapeutic contexts are increasingly used across a much wider range of contexts and practices.^5^ This can be problematic because the basis of psychotherapeutic work is an individual's own internal thoughts and feeling states. While it is widely acknowledged that an individual has privileged access to these, it is also widely accepted that there is not usually an equal distribution of clinical knowledge between a healthcare professional and their patient or client. Indeed, this was one of the key features of Balint's description of the ‘lop‐sided’ relationship between doctor and patient. A person's expert status in talking about their feelings cannot be straightforwardly transferred to understanding their symptoms, for example. This is one reason why using the terms interchangeably is problematic; another is that personhood as a philosophical concept used by authors such as Kitwood,^6^ and patienthood as a practical one (e.g., in an acute care appointment), are not easily or straightforwardly substitutable by simply swapping one word for another in an otherwise unchanged policy context.

## 4 WHAT IS THE EVIDENCE FOR PATIENT‐CENTRED CARE?

The widespread adoption of PCC in NHS policy for service delivery might suggest a strong evidence base. However, examining the empirical evidence for the effectiveness of PCC tells a different story. While there are individual studies which report positive impacts, wider research (including Cochrane systematic reviews of PCC interventions) does not show a clear link between the adoption of PCC in a setting, and a corresponding improvement in health outcomes.^7^ Some reviews have been able to demonstrate increased patient satisfaction where PCC is practised, but even this is not universally true. The only consistent finding is a circularity: that where practitioners are trained to use a particular PCC intervention, this increases the practice of PCC as measured by that specific intervention. However, this lack of evidence for the impact of PCC has not prompted a more critical re‐evaluation. Instead, the problem is usually laid at the door of professionals, with an assumption that if only we could give them more or ‘better’ training in PCC, we would obtain the missing evidence.

## 5 WHY DOESN'T PCC WORK IN PRACTICE?

As I have shown, the pervasiveness of PCC is not grounded in empirical evidence. Instead, it is based on a moral position that makes intuitive sense. But I analysed a large corpus of audio and video recorded healthcare interactions collected over a 25‐year period from a wide range of healthcare settings that were underpinned by a commitment to practise PCC.^8^ In all of these settings I had observed that attempts to practice PCC sometimes ran into difficulties, and I wanted to understand why. Examining these interactions as they actually unfold on wards, clinics and consulting rooms shows that there is not generally the struggle for control that PCC assumes. PCC is underpinned by the language of patient autonomy and choice, but a focus on control as a property that rests with only one or the other party can obscure the way that choice and control are negotiated and constructed collaboratively. The issue that I came across repeatedly in my data was that, if choice and control are seen as properly belonging exclusively to patients, there is no longer any clear place for medical expertise in healthcare decision making. There are two potential consequences of this, and both are problematic for patients. The first is that medical decisions can become cast as purely private matters that patients must deal with alone, based on how they ‘feel’ about the options or how much they ‘worry’ about the alternatives. Whilst such an approach undoubtedly preserves patient autonomy, it does not necessarily bring about the empowerment promised by PCC. Instead, it can result in patients feeling abandoned, and trying to elicit medical advice indirectly, through questions such as ‘What would you do in my situation?’ or ‘What do most people do?’.

The second potential consequence of giving control to the patient‐ and perhaps its logical end point, if this is assumed to be the ultimate aim of PCC‐ is in practices of affirmative care. From an affirmative care perspective, the professional's role is to empathetically support the assertions of the client, and client understandings of their situation are not to be challenged or questioned. Sociologically speaking, this kind of approach has its roots in a wider cultural movement where the revelation of inner experience leads inexorably and unproblematically to truth or authenticity.^9^ However, in practical terms, it officially removes dimensions of the resources that professionals might otherwise bring to bear in healthcare consultations, such as their knowledge of how different courses of action have impacted different patients in different contexts. As Hilary Cass's current UK inquiry into the provision of gender identity services for under 18s has highlighted, the end point of this approach may potentially be in practices that do not meet care standards.

## 6 WHAT IS THE ALTERNATIVE?

I argue that there are two things that we need to make happen. We need to begin by recognizing the difference between medical *expertise* (meaning the right to knowledge in a particular area) and medical *authority* (meaning the right to decide what should happen based on that knowledge). PCC has rightly highlighted that medical authority can be problematic, in rejecting the ‘doctor knows best’ attitude of unilateral medical paternalism. Successive investigations into high profile medical scandals, such as the Francis Inquiry in the United Kingdom, have shown the role that a culture of unchallenged medical authority can play in these, and the need to address this. However, with its emphasis on choice and control, PCC has inadvertently problematised medical expertise as well. All the evidence from my data shows that medical expertise is important to patients; a large part of why they consult with a healthcare professional in the first place is because they don't treat all sources of healthcare information as equal, and they lack the knowledge, or the ability to apply that knowledge, to solve their own problems. This suggests that instead of continuing existing training endeavours in the hope that professionals will practice ‘better’ PCC, it would be more fruitful to recognize that professionals are sources of knowledge that patients both want and need, and to think about how we can re‐centre medical expertise in the practice of contemporary healthcare in ways that are productive for and acceptable to patients. This does not mean that patient expertise is not important‐ far from it‐ but it also means we need to think about how this can be best elicited, incorporated and utilized. Rather than using different tools to score the extent to which individual consultations allow patients to express this (and in the knowledge that different PCC measurement tools have been shown to produce quite different results for the same consultations), we need to shift our focus to how this patient expertise can be incorporated on a wider and more fundamental level. Co‐design of services and co‐production of healthcare resources are important ways in which the central importance of patient perspectives and experience can be recognized and incorporated in a collaborative rather than conflict‐based model of healthcare.

The second thing we need to do is to recognize that, wherever patient‐facing healthcare policies are formulated, most will depend on being talked into existence at the point of care delivery. This means that, without an understanding of how healthcare interaction works in practice, they are potentially set up to fail. PCC is founded in a moral position, rather than empirical evidence, but as the constant search for ‘better’ training in an attempt to evidence an impact on healthcare outcomes shows, the problem with this is that it becomes very difficult to step outside the moral shelter of the position, even in the face of contradictory evidence. If control is simplistically conceptualized as a consumerist property, then it belongs only to one or other party, but in real‐life healthcare interaction it is negotiated and constructed collaboratively. Studying healthcare delivery as it happens shows us how practices that we might imagine will promote patient empowerment, or even those that might work to promote empowerment in other settings, often don't function this way in healthcare. It is common for interaction to be studied as part of post hoc‐policy evaluation. However, the example of PCC shows the need for an understanding of interaction being used to inform healthcare policy making, rather than simply using it to judge the success or failure of these policies after their implementation.

## CONFLICT OF INTEREST STATEMENT

The author declares no conflict of interest.

## Data Availability

This is a Viewpoint article drawing on a range of previous research and so data sharing is not applicable.
